# Graphene quantum dots blocking the channel egresses of cytochrome P450 enzyme (CYP3A4) reveals potential toxicity

**DOI:** 10.1038/s41598-023-48618-z

**Published:** 2023-11-30

**Authors:** Yuqi Luo, Jinjun Li, Zonglin Gu, Yaoxing Huang

**Affiliations:** 1 Department of Gastrointestinal and Hepatobiliary Surgery, Shenzhen Longhua District Central Hospital, No. 187, Guanlan Road, Longhua District, Shenzhen, 518110 Guangdong Province China; 2 College of Physical Science and Technology, Yangzhou University, Jiangsu, 225009 China; 3 Department of Gastroenterology, Guangzhou First People’s Hospital, School of Medicine, South China University of Technology, Guangzhou, 510180 Guangdong Province China

**Keywords:** Biophysics, Nanoscience and technology

## Abstract

Graphene quantum dots (GQDs) have garnered significant attention, particularly in the biomedical domain. However, extensive research reveals a dichotomy concerning the potential toxicity of GQDs, presenting contrasting outcomes. Therefore, a comprehensive understanding of GQD biosafety necessitates a detailed supplementation of their toxicity profile. In this study, employing a molecular dynamics (MD) simulation approach, we systematically investigate the potential toxicity of GQDs on the CYP3A4 enzyme. We construct two distinct simulation systems, wherein a CYP3A4 protein is enveloped by either GQDs or GOQDs (graphene oxide quantum dots). Our results elucidate that GQDs come into direct contact with the bottleneck residues of Channels 2a and 2b of CYP3A4. Furthermore, GQDs entirely cover the exits of Channels 2a and 2b, implying a significant hindrance posed by GQDs to these channels and consequently leading to toxicity towards CYP3A4. In-depth analysis reveals that the adsorption of GQDs to the exits of Channels 2a and 2b is driven by a synergistic interplay of hydrophobic and van der Waals (vdW) interactions. In contrast, GOQDs only partially obstruct Channel 1 of CYP3A4, indicating a weaker influence on CYP3A4 compared to GQDs. Our findings underscore the potential deleterious impact of GQDs on the CYP3A4 enzyme, providing crucial molecular insights into GQD toxicology.

## Introduction

Carbon-based nanomaterials (CBNs) have emerged as a significant research focus in recent decades since original discoveries such as fullerene C_60_ in 1985^1^, carbon nanotubes (CNTs) in 1991^2^, and graphene in 2004^3^. These discoveries have sparked immense interest due to the exceptional and distinctive properties inherent to CBNs, including high specific surface area, size and dimensional effects, structural versatility, and superior mechanical, electrical, and optical characteristics^4–10^. Consequently, CBNs have garnered considerable attention across various scientific communities and found versatile applications as gas storage devices, transistors, sensors, nanocarriers, and nanodrugs^11–15^. In particular, extensive efforts have been devoted to exploring the application of CBNs in the biomedical field^6,16–25^. However, researchers must carefully consider the potential toxicity of CBNs prior to their formal utilization^26,27^. For example, graphene exhibits severe toxicity towards certain biomolecules. Tu et al.^28^ reported robust insertion of graphene and graphene oxide into cellular membranes, leading to membrane lipid extraction and cell death. Various studies have demonstrated that graphene can disrupt the structural integrity, including the secondary and tertiary structures, of proteins, resulting in protein toxicity^29^. Luan and co-workers showed that graphene disrupts signal transduction by interfering with physiological protein–protein interactions^30^. Additionally, graphene can alter the helical conformation and base pairs of double-stranded DNA, potentially causing genotoxicity^31^.

Thanks to their small sizes and quantum effects, graphene quantum dots (GQDs) have garnered significant attention across various fields^32–35^. Remarkably, three scientists were awarded the 2023 Nobel Prize in Chemistry for their groundbreaking discoveries and synthesis of quantum dots. Predictably, GQDs will become a research hotspot in the near future. In organisms, the biocompatibility of GQDs is a significant factor for their further application^36,37^. Chong et al.^38^ experimentally investigated the in vitro and in vivo toxicity of GQDs using AFM, TEM, FTIR, XPS, and elemental analysis. Their results confirmed that GQDs exhibit very low cytotoxicity owing to their ultra-small size and high oxygen content. Similarly, Chu and colleagues^39^ explored the effects of GQDs on reproductive and offspring health in mammals and found that GQD exposure via oral gavage or intravenous injection had no effect on the frequency and timing of sexual behaviors in male mice. Furthermore, Xu and co-workers^40^ demonstrated that luminescent GQDs show weak toxicity to HeLa cells and zebrafish embryos. However, Das et al.^41^ emphasized that GQD toxicity depends on various factors such as size, concentration, surface chemistry, and doping. In particular, some studies have highlighted potential toxic effects, including dopaminergic neurodegeneration, induction of apoptosis, autophagy, and inflammatory responses^42,43^. Thus, supplementing the toxicity profile of GQDs is critical to comprehensively understand the biosafety of GQDs.

In this study, we employ molecular dynamics simulations to investigate the potential influence of GQDs and GOQDs on the CYP3A4 protein. The members of the Cytochrome P450 (CYP) superfamily are pivotal in facilitating essential monooxygenation reactions, playing a critical role in the phase I metabolism of diverse endogenous and exogenous compounds, encompassing pharmaceuticals and xenobiotics^44^. Notably, CYPs are central to drug metabolism^45^, with over 90% of pharmaceutical compounds undergoing metabolic transformation primarily mediated by a select set of five significant CYP members. Of these, human CYP3A4 stands out by metabolizing approximately half of the therapeutically utilized drugs^46^. The functional roles of channels within CYP3A4 are of paramount importance, providing a conduit for ligands to ingress and products to egress from the deeply embedded active site. Considering the significance and abundance distributions of CYP3A4 in human body (e.g., the liver and the small intestine)^47^, we try to explore the potential influence of GQDs and GOQDs on CYP3A4. Our simulations demonstrate that GQDs can make direct contact with the bottleneck residues of Channels 2a and 2b. Additionally, GQDs can cover the egresses of Channels 2a and 2b, suggesting the potential blockage of these channels by GQDs. In contrast, we observe only partial coverage of one GOQD in the vicinity of Channel 1. Therefore, GOQDs exhibit weaker toxicity towards CYP3A4 than GQDs. Overall, our results shed light on the potential toxicity of GQDs towards CYP3A4, providing valuable molecular insights.

## Results

**Figure 1:**
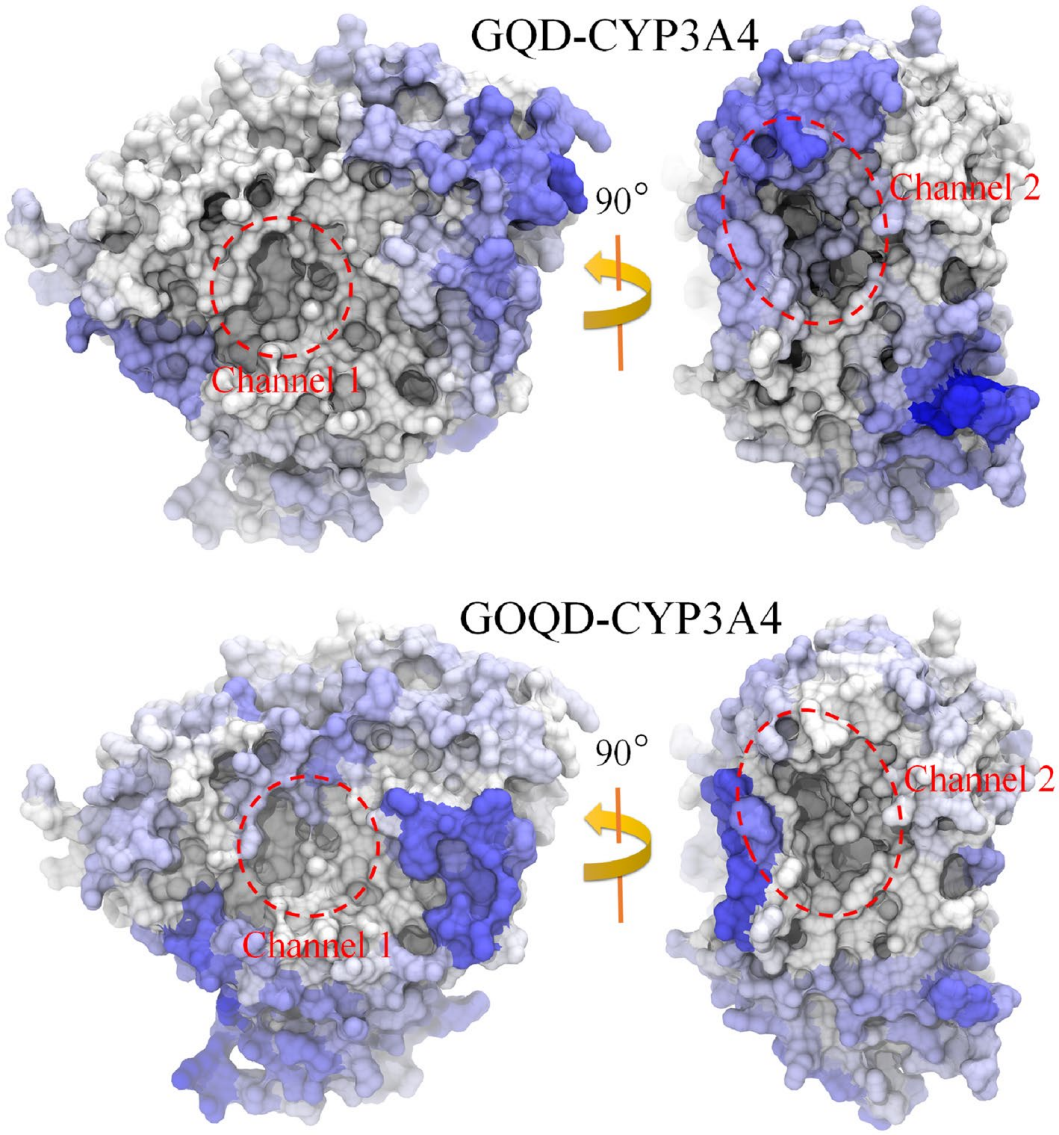
Maps of contact probabilities of GQD (upper) and GOQD (bottom) binding to CYP3A4 protein. Blue and white surfaces indicate high and low contact probabilities, respectively. The egresses of Channel 1 and 2 are highlighted by red dashed circles. All the figures are generated by VMD software package^56^ (http://www.ks.uiuc.edu/Research/vmd/).

We constructed two typical simulation systems as illustrated in Figure S1. In each system, ten GQD or GOQD dots were separately dispersed around CYP3A4, resulting in two simulation systems. Subsequently, each system was performed for 5 parallel trajectories, each with a duration of 100 ns. The resulting simulation conformations were presented in Figure S2 (depicting GQD/CYP3A4 interaction) and S3 (depicting GOQD/CYP3A4 interaction). Initially, we summarized the contact between GQD and GOQD with the CYP3A4 surface. As demonstrated in Fig. 1, the contact isosurface illustrates the contact probability of CYP3A4 by GQDs or GOQDs, with the blue region denoting a high probability of contact. The contact probability was averaged from all five parallel trajectories of each system. We note that there are many holes distributed on the CYP3A4 surface, which are caused by the fluctuant and irregular protein surface. CYP3A4 surface contains channel egresses (Figure S4) that are aisles connecting the outside environment and the enzyme interior and that allow exogenous compounds to enter and undergo metabolism, underscoring the vital role of channels in CYP3A4 for ligand access and product egress from the buried active site. CYP3A4 typically encompasses some channel egresses, such as Channel 1 and Channel 2, as depicted in Fig. 1. Channel 2 egresses primarily comprise Channel 2a, 2b, and 2c (also see Figure S4). Additionally, in these channels, specific residues (i.e., bottleneck residues) regulate the permeability of each channel^48^, e.g., R105, R106, S119, and I120 for Channel 1, R106, and P107 for Channel 2b, L216 and L221 for Channel 2a, and F113 for Channel 2c. The particular bottleneck residues determine the potential openings for ligand entry or egress^49^. Remarkably, we observe a high contact probability of GQD around Channel 2 egresses, suggesting the potential blockage of GQD in Channel 2 of CYP3A4.

To delve into the contact details, we further summarized the binding probabilities of GQD to the bottleneck residues in each channel (Table 1). Clearly, most bottleneck residues exhibited very low contact probabilities with GQD, primarily because they are typically buried within each channel. However, P107 in Channel 2b and L221 in Channel 2a presented relatively high contact probabilities, with corresponding values of 0.118 and 0.178. The bottleneck residues can be categorized into three types, i.e., hydrophobic (I120, P107, L216, L221 and F113), hydrophilic (S119) and basic (R105 and R106) residues. We note that hydrophobic and basic residues separately occupy 62.5% and 25% among the whole bottleneck residues, whereas hydrophilic residue only takes up 12.5%. Interestingly, previous studies have demonstrated that hydrophobic residues can be easily adsorbed onto graphene surface through hydrophobic interaction^50^, and that basic residues also prefer to adhere to graphene surface due to the vdW interaction^51^. Therefore, the specificity of dominant hydrophobic and basic residues in bottleneck residues make them susceptible to interaction with GQDs. These findings imply the probable influence of GQD on Channel 2b and Channel 2a.

**Table 1:** Binding probabilities of GQD to some bottleneck residues in each channel.

Channel 1	R105	R106	S119	I120
	0.008	0.056	0	0
Channel 2b	R106	P107		
	0.056	0.188		
Channel 2a	L216	L221		
	0.026	0.178		
Channel 2c	F113			
	0			

**Figure 2:**
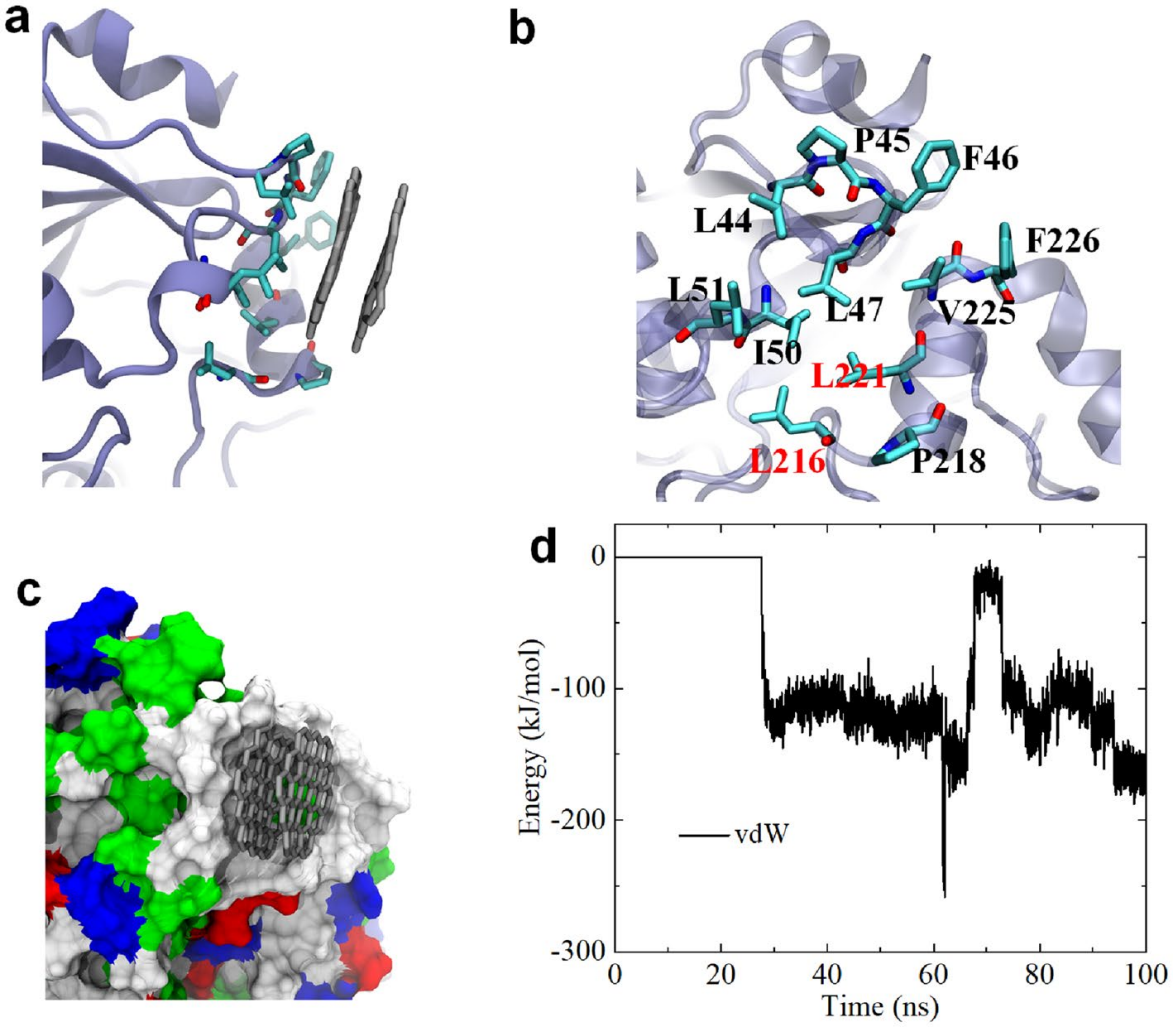
(**a**) GQDs binding to the egress of Channel 2a. GQDs are shown with gray sticks; CYP3A4 is displayed by iceblue ribbon; and critical residues around Channel 2a are shown by cyan (carbon), red (oxygen) and blue (nitrogen) sticks. (**b**) Local illustration of critical residues associating to GQDs binding to Channel 2a egress. L216 and L221 are bottleneck residues of Channel 2a. (**c**) GQDs binding to the egress of Channel 2a. CYP3A4 is shown with colored surfaces. White, green, red and blue surfaces separately represent hydrophobic, hydrophilic, acidic and basic residues. (**d**) Interaction energy (i.e., vdW energy) evolution of these two GQDs and CYP3A4. All the figures are generated by VMD software package^56^ (http://www.ks.uiuc.edu/Research/vmd/).

For a more detailed understanding of the binding characteristics around P107 and L221 in CYP3A4, we plotted the binding conformations, as depicted in Fig. 2. We observed that the binding at the Channel 2a position (also depicted in Figure S2a) occurred between two stacked GQDs and certain residues. We then highlight the bound residues, as shown in Fig. 2b. These residues include L44, P45, F46, L47, I50, L51, L216, P218, L221, V225, and F226. Remarkably, both L216 and L221 are bottleneck residues associated with Channel 2a, indicating the potential effect of GQD on the permeability of Channel 2a. Additionally, Figure S5 displays the contact probabilities of these residues, and the contact probabilities are mostly not too low, further supporting the high affinity between GQD and Channel 2a egress. Intriguingly, all these contacted residues are completely hydrophobic. Considering the hydrophobic nature of GQD, the binding of GQD to Channel 2a egress is likely driven by hydrophobic interactions. We further presented the CYP3A4 surface around the binding region in Channel 2a egress (Fig. 2c), where hydrophobic, hydrophilic, acidic, and basic residues are depicted in white, green, red, and blue, respectively. Clearly, the Channel 2a egress is almost covered by white surface, indicating that Channel 2a egress has a strong hydrophobicity. The two adsorbed GQDs are entirely surrounded by a white surface, confirming again the significant role of hydrophobic interactions in driving the binding of GQD to Channel 2a egress. It should be noted that the hydrophobic residues usually comprise hydrophobic segments at their sidechains, e.g., hydrocarbon chains and aromatic rings. These hydrophobic segments can form hydrophobic interaction with GQDs. Additionally, it is important to highlight the involvement of two aromatic residues (F46 and F226) in the interfacial adhesion of the two GQDs to the Channel 2a egress. Aromatic residues are known to involve in the strong π–π stacking interactions with graphene^51^, making the π–π interaction, another significant driving force for the binding of GQDs to Channel 2a egress. Moreover, we calculated the interaction energy between these two GQDs and CYP3A4 (Fig. 2d), revealing a substantial contribution from van der Waals (vdW) interaction to the binding of GQD to Channel 2a egress. Consequently, according to a previous study^52^, the interaction mechanism of GQDs and CYP3A4 is that the interplay of hydrophobic, π–π stacking and vdW interactions drive the interaction of GQDs to the Channel 2a egress of CYP3A4 surface, finally inducing blockage of Channel 2a egress.

**Figure 3:**
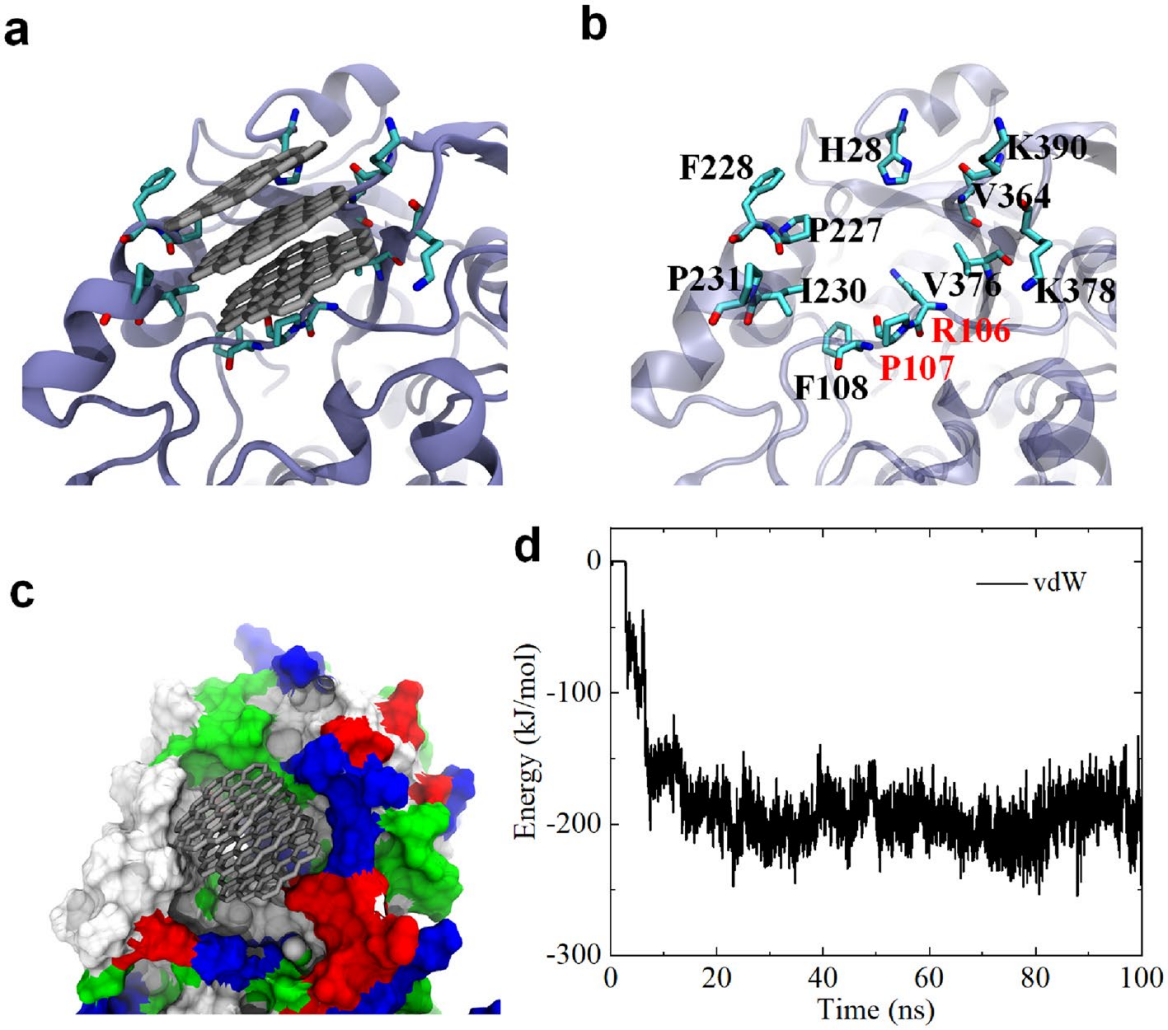
(**a**) GQDs binding to the egress of Channel 2b. GQDs are shown with gray sticks; CYP3A4 is displayed by iceblue ribbon; and critical residues around Channel 2b are shown by cyan (carbon), red (oxygen) and blue (nitrogen) sticks. (**b**) Local illustration of critical residues associating to GQDs binding to Channel 2a egress. R106 and R107 are bottleneck residues of Channel 2b. (**c**) GQDs binding to the egress of Channel 2b. CYP3A4 is shown with colored surfaces. White, green, red and blue surfaces separately represent hydrophobic, hydrophilic, acidic and basic residues. (**d**) Interaction energy (i.e., vdW energy) evolution of these two GQDs and CYP3A4. All the figures are generated by VMD software package^56^ (http://www.ks.uiuc.edu/Research/vmd/).

Subsequently, we investigated the binding of GQDs to Channel 2b (as depicted in Fig. 3a). We observed that the binding at the Channel 2b position (also shown in Figure S2a) occurred between three stacked GQDs and specific residues. We then highlight the bound residues, as presented in Fig. 3b. These residues include H28, R106, P107, F108, P227, F228, I230, P231, V364, V376, K378, and K390. Notably, both R106 and P107 are bottleneck residues associated with Channel 2b, indicating the potential effect of GQD on Channel 2b permeability. The contact residues can be classified into two types: hydrophobic residues (including P107, F108, P227, F228, I230, P231, V364, and V376) and basic residues (including H28, R106, K378, and K390). The hydrophobic residues occupy 66.7% among the total contact residues, hinting that the hydrophobic interaction contributes to the adsorption of GQDs to Channel 2b egress. Also, two aromatic residues (F108 and F228) involve the adsorption of GQDs to Channel 2b egress, suggesting the contribution of π–π stacking interaction. In addition, basic residues also have a ratio of 33.3%. As previously reported^51^, basic residues can exhibit strong interactions with graphene due to their long side chains capable of generating strong vdW interactions with the graphene sheet. Thus, these results suggest that the adsorption of GQDs to the Channel 2b egress is primarily driven by hydrophobic, π–π stacking and vdW interactions. We further illustrated the CYP3A4 surface around the binding region in Channel 2b egress (Fig. 3c), where the three adsorbed GQDs are surrounded by white (hydrophobic residues), blue (basic residues), and green (hydrophilic residues) surfaces. The white and blue surfaces occupy the majority of the contact area, confirming once again the significant role of hydrophobic and vdW interactions in driving the binding of GQD to Channel 2b egress. To elucidate the role of vdW interaction, we calculated the interaction energy between these three GQDs and CYP3A4 (Fig. 3d). Clearly, the vdW energy surpasses − 200 kJ/mol, affirming its critical contribution to the binding of GQD to Channel 2b egress. Therefore, according to a previous study^52^, the interaction mechanism of GQDs and CYP3A4 is that the interplay of hydrophobic, π–π stacking and vdW interactions drive the interaction of GQDs to the Channel 2a egress of CYP3A4 surface, inducing blockage of Channel 2b egress.

**Figure 4:**
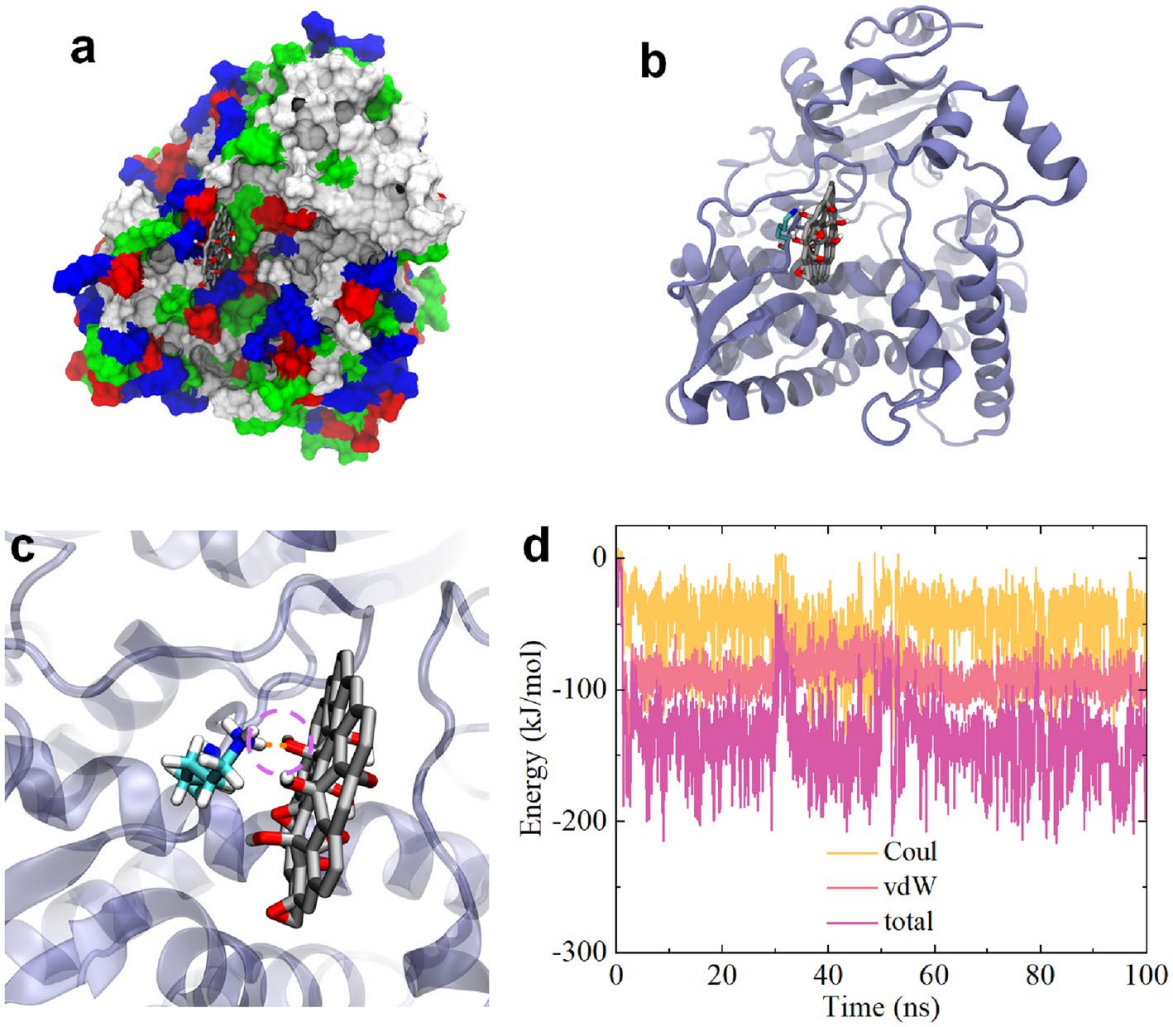
(**a**) GOQD binding to the egress of Channel 1. GOQD is shown with gray (carbon), red (oxygen) and white (hydrogen) sticks. CYP3A4 is shown with colored surfaces. White, green, red and blue surfaces separately represent hydrophobic, hydrophilic, acidic and basic residues. (**b**) GOQD binding to the K487 near Channel 1 of CYP3A4. CYP3A4 is displayed by iceblue ribbon; and the critical residue K487 around Channel 1 are shown by cyan (carbon) and blue (nitrogen) sticks. (**c**) Local illustration of hydrogen bond formed by K487 and GOQD. The hydrogen bond is shown with orange dash line, which is highlighted by magenta dashed circle. (**d**) Interaction energies, including vdW, Coulomb (Coul) and total energy evolutions of GOQD binding to Channel 1 egress of CYP3A4. All the figures are generated by VMD software package^56^ (http://www.ks.uiuc.edu/Research/vmd/).

The binding probabilities of GOQD to the bottleneck residues of CYP3A4 are summarized in Table 2. These binding probabilities exhibit very low values, indicating that GOQD has a low probability of blocking the channel egresses of CYP3A4. Upon evaluating the final binding conformations (Figure S3), we observed a potential partial blockage of GOQD to Channel 1 (Figure S3d). As illustrated in Fig. 4a, one GOQD adsorbs in the vicinity of the Channel 1 egress, although it does not directly interact with the bottleneck residues. Further analyses revealed that one basic residue, K487, intimately interacts with the GOQD surface (Fig. 4b) and forms a hydrogen bond with an oxygen group on GOQD (Fig. 4c). The interaction energy calculations (Fig. 4d) confirmed that the binding of this GOQD to Channel 1 egress is mediated by both vdW and Coulomb energies. Consequently, based on a previous study^52^, the interaction mechanism of GOQDs is that the interplay hydrogen bonding, vdW, and Coulomb interactions drive the interaction of GOQDs to the CYP3A4 surface, potentially leading to weak toxicity and partial blockage of Channel 1 permeability in CYP3A4. However, compared to GQD, GOQD exhibits a lower capacity to impact or block CYP3A4 channels.

**Table 2:** Binding probabilities of GOQD to some bottleneck residues in each channel.

Channel 1	R105	R106	S119	I120
	0.02	0.022	0	0.004
Channel 2b	R106	P107		
	0.022	0.022		
Channel 2a	L216	L221		
	0	0.066		
Channel 2c	F113			
	0.036

## Conclusion

In summary, this study investigates the potential impact of GQD and GOQD on the CYP3A4 enzyme utilizing a molecular dynamics simulation approach. Two representative simulation systems are constructed, each comprising a CYP3A4 surrounded by ten GQDs or GOQDs. The results demonstrate that GQDs, forming clusters of two or three entities, adhere to and obstruct the egresses of Channel 2a and 2b of CYP3A4. Importantly, GQDs can make direct contact with the bottleneck residues of Channel 2a and 2b, indicating a potential adverse influence of GQDs on these two channels. Our calculations affirm that the adsorption of GQDs to Channel 2a and 2b is facilitated by a combined effect of hydrophobic, π–π stacking and vdW interactions. Conversely, only one trajectory reveals a partial blockage of one GOQD to Channel 1, suggesting that GOQD has a weaker influence on CYP3A4 compared to GQD. These findings elucidate the potential toxicity of GQDs towards the CYP3A4 enzyme, providing valuable insights for the safe and effective utilization of such nanomaterials in biomedicine.

## Methods

We conducted two representative simulations: one involving a CYP3A4 protein and ten GQDs, and the other comprising a CYP3A4 protein and ten GOQDs. The initial placement of GQDs and GOQDs around CYP3A4 ensured an initial distance greater than 1.2 nm, preventing any artificial contact between the GQD/GOQD and CYP3A4. GQDs were simulated using Lennard–Jones (LJ) particles with parameters set at ε_c_ = 0.36 kJ/mol and σ_c_ = 0.34 nm. The GOQDs were prepared based on established parameters from a previous study^53^. Both GQD and GOQD (Figure S6) were designed to have an equivalent surface dimension. GQD and GOQD have the same basal area and carbon structure, whereas GOQD was totally coated by 5 hydroxy groups and 5 epoxy groups. The crystal structure of human CYP3A4 (PDB ID: 1TQN) served as the basis for initializing all protein configurations, consistent with prior research^50^. Subsequently, the GQDs/CYP3A4 and GOQDs/CYP3A4 complexes were dissolved in 0.15 M NaCl solutions.

All simulations were carried out with the GROMACS (version 2018) software package^54^ using the CHARMM27 force field^55^. The VMD software^56^ was used to analyze and visualize the simulation results. The TIP3P water model^57^ was adopted to treat the water molecules since it is widely used to investigate the interaction between nanomaterials and biomolecules. The Lennard–Jones (LJ) potential was calculated according to the formula: $V(r)=4\varepsilon \left[{\left(\frac{\sigma }{r}\right)}^{12}-{\left(\frac{\sigma }{r}\right)}^{6}\right]$, wherein $V(r)$ indicates the LJ potential energy, *r* denotes the distance of the pairwise atoms. The *ε* equals to $\sqrt{{\varepsilon }_{i}{\varepsilon }_{j}}$ and the *σ* equals to $\frac{{\sigma }_{i}{+\sigma }_{j}}{2}$, wherein ${\varepsilon }_{i}$, ${\varepsilon }_{j}$ and ${\sigma }_{i}$, ${\sigma }_{j}$ were two critical LJ parameters for the calculated atoms *i* and *j*. The temperature was maintained at 300 K using a v-rescale thermostat^58^ and pressure was kept at 1 atm using Parrinello–Rahman barostat^59^. The long-range electrostatic interactions were treated with the PME method^60^, and the van der Waals (vdW) interactions were calculated with a cutoff distance of 1.2 nm. All solute bonds associated with hydrogen atoms were maintained constant at their equilibrium values with the LINCS algorithm^61^, and water geometry was also constrained using the SETTLE algorithm^62^. During the production runs, a time step of 2.0 fs was used, and data were collected every 10 ps. Each system was investigated for five independent 100 ns trajectories.

### Supplementary Information

Supplementary Figures.

## Data Availability

The datasets used and/or analysed during the current study available from the corresponding author on reasonable request.
