# Supplementary material for: Graphene quantum dots blocking the channel egresses of cytochrome P450 enzyme (CYP3A4) reveals potential toxicity
Source: Sci Rep. 2023 Nov 30;13:21091. doi: 10.1038/s41598-023-48618-z (PMC10689800; doi:10.1038/s41598-023-48618-z)
Supplement: Supplementary file 1 — Supplementary Figures. [file 41598_2023_48618_MOESM1_ESM.docx]

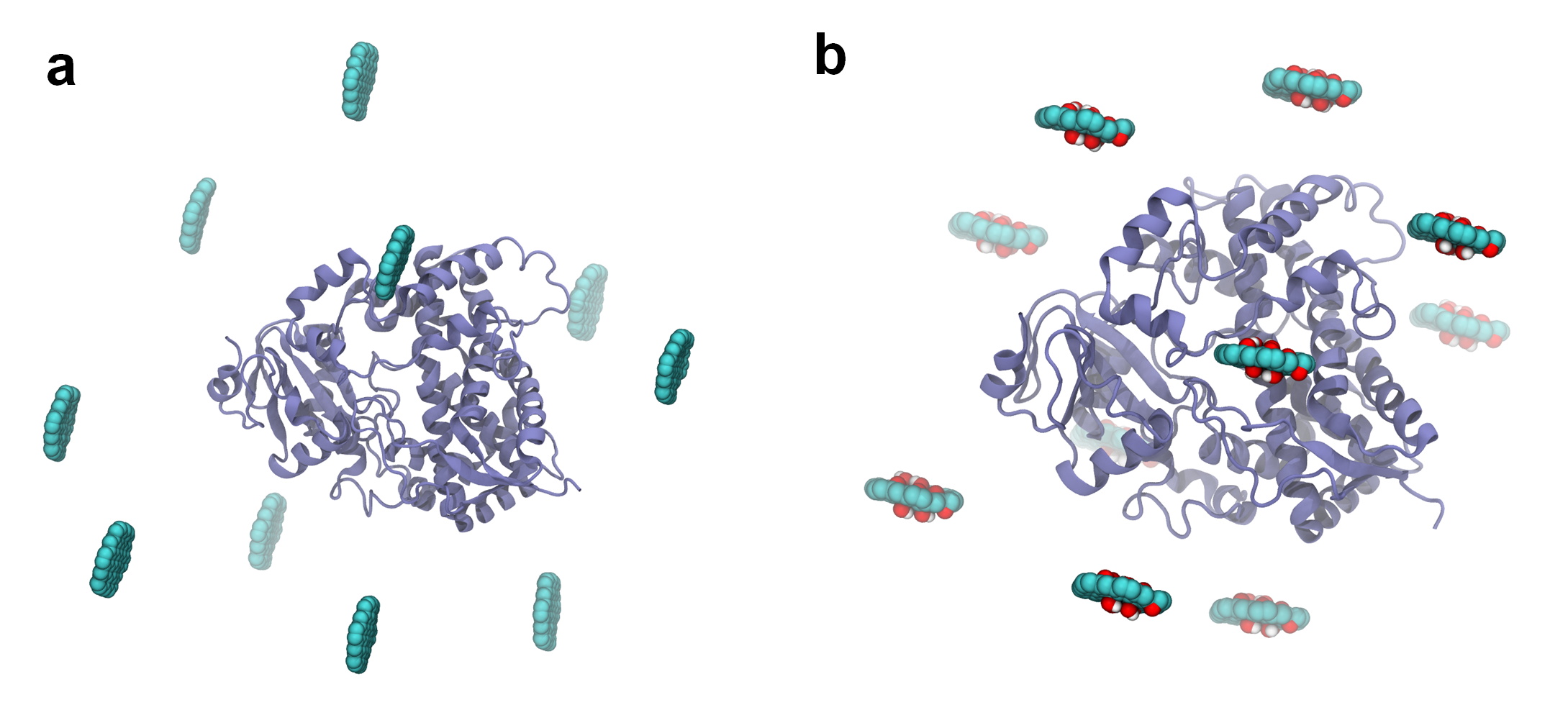


Figure S1. Initial setups of GQD/CYP3A4 (a) and GOQD/CYP3A4 (b). All the figures are generated by VMD software package (http://www.ks.uiuc.edu/Research/vmd/, J. Mol. Graph. Model. 1996, 14 (1), 33-38).


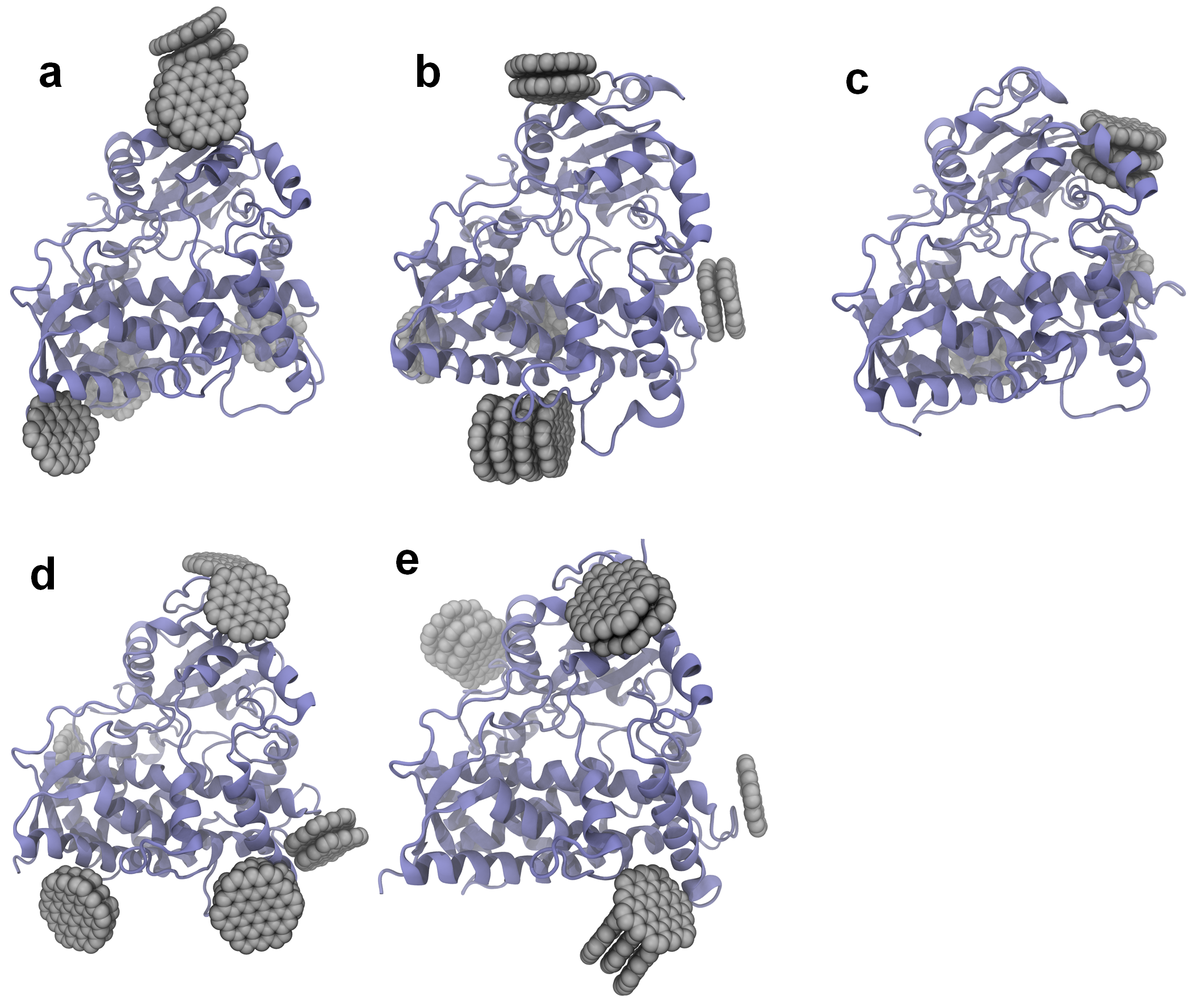


Figure S2. Final conformations of GQD binding to CYP3A4 in five parallel simulations. All the figures are generated by VMD software package (http://www.ks.uiuc.edu/Research/vmd/, J. Mol. Graph. Model. 1996, 14 (1), 33-38).


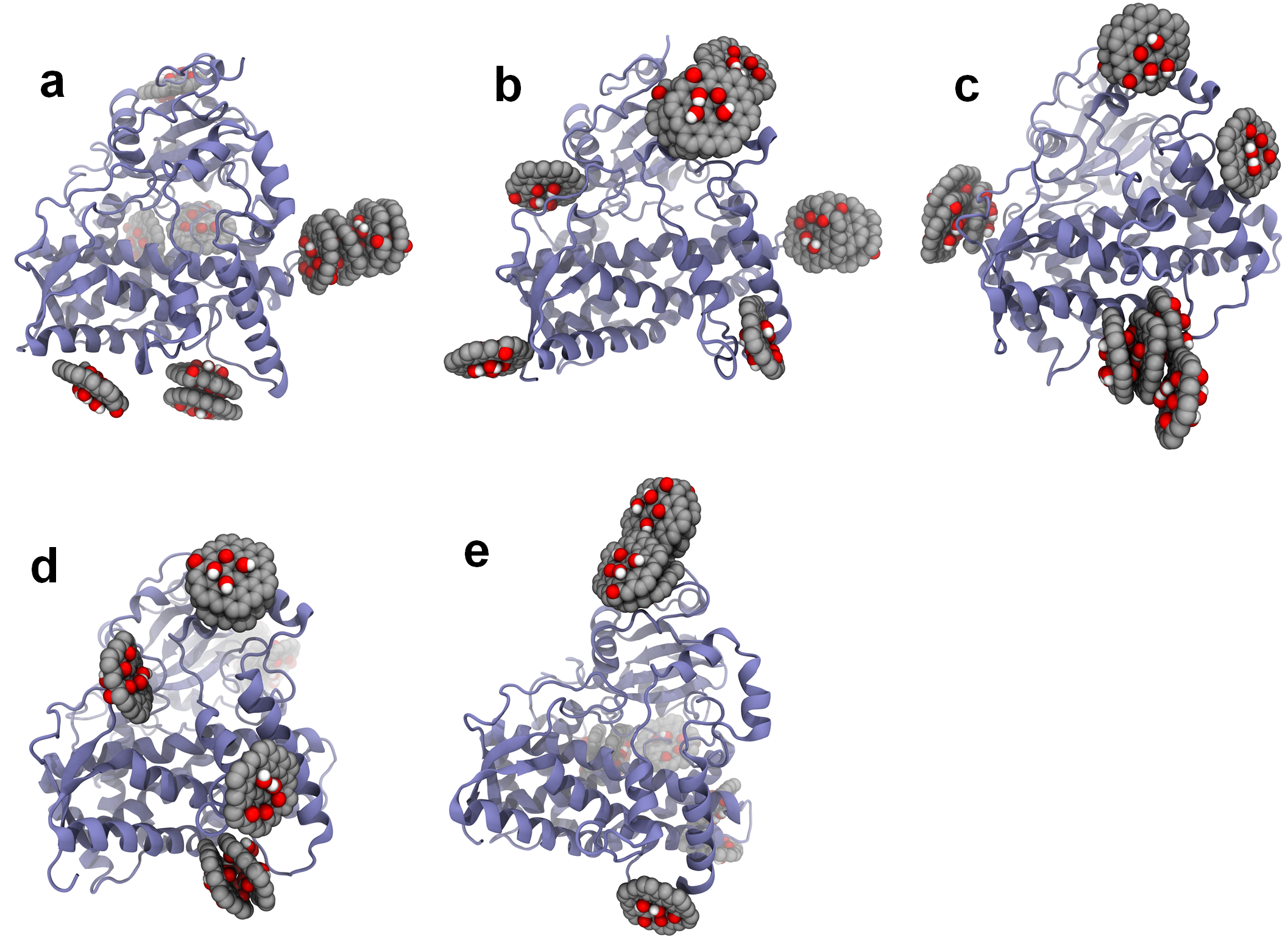


Figure S3. Final conformations of GOQD binding to CYP3A4 in five parallel simulations. All the figures are generated by VMD software package (http://www.ks.uiuc.edu/Research/vmd/, J. Mol. Graph. Model. 1996, 14 (1), 33-38).


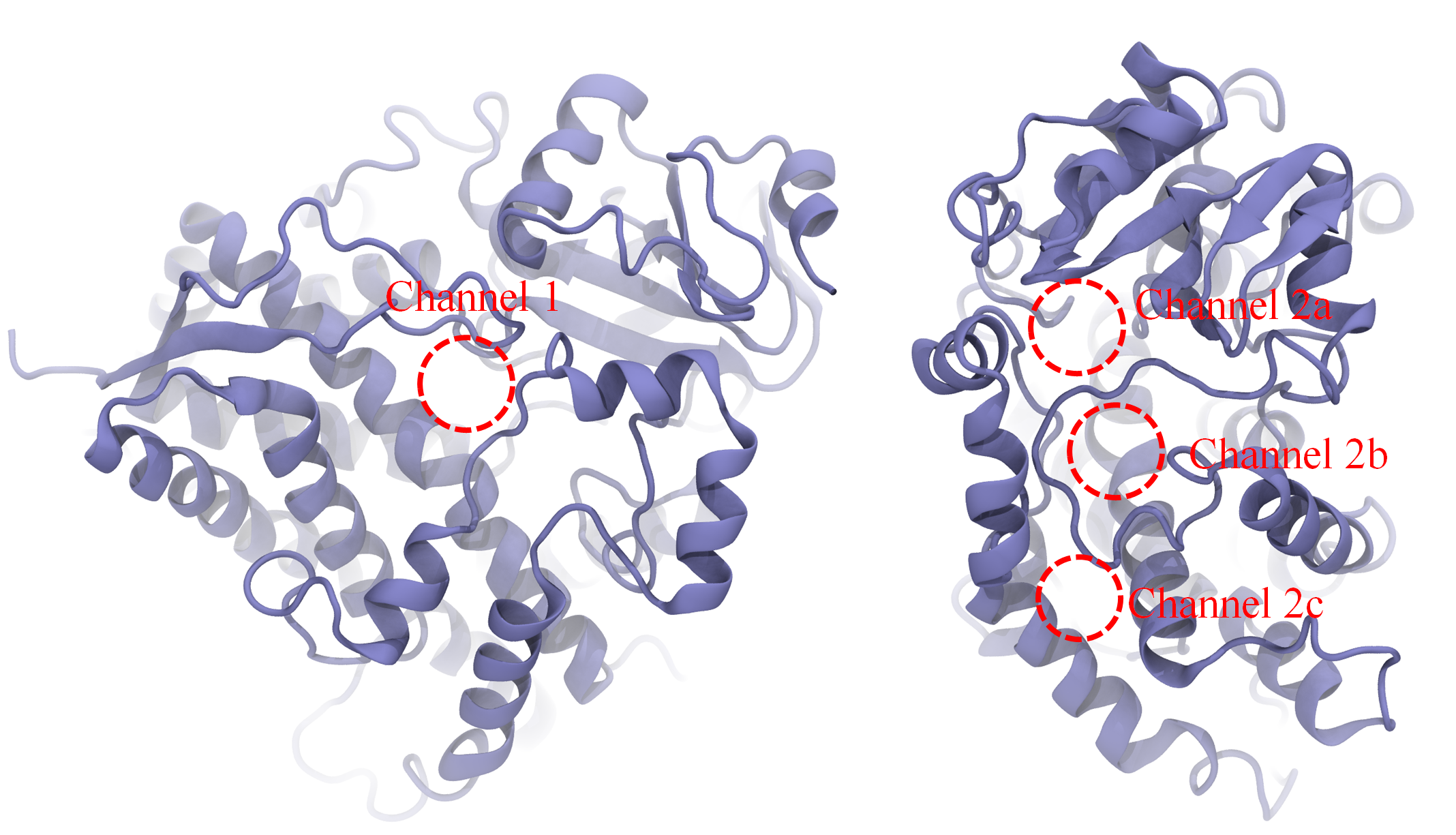


Figure S4. Illustrations of the egresses of each channel. The configurations of CYP3A4 herein are in accordance with the pictures in Figure 1. The channel egresses are highlighted by red dashed circles. All the figures are generated by VMD software package (http://www.ks.uiuc.edu/Research/vmd/, J. Mol. Graph. Model. 1996, 14 (1), 33-38).


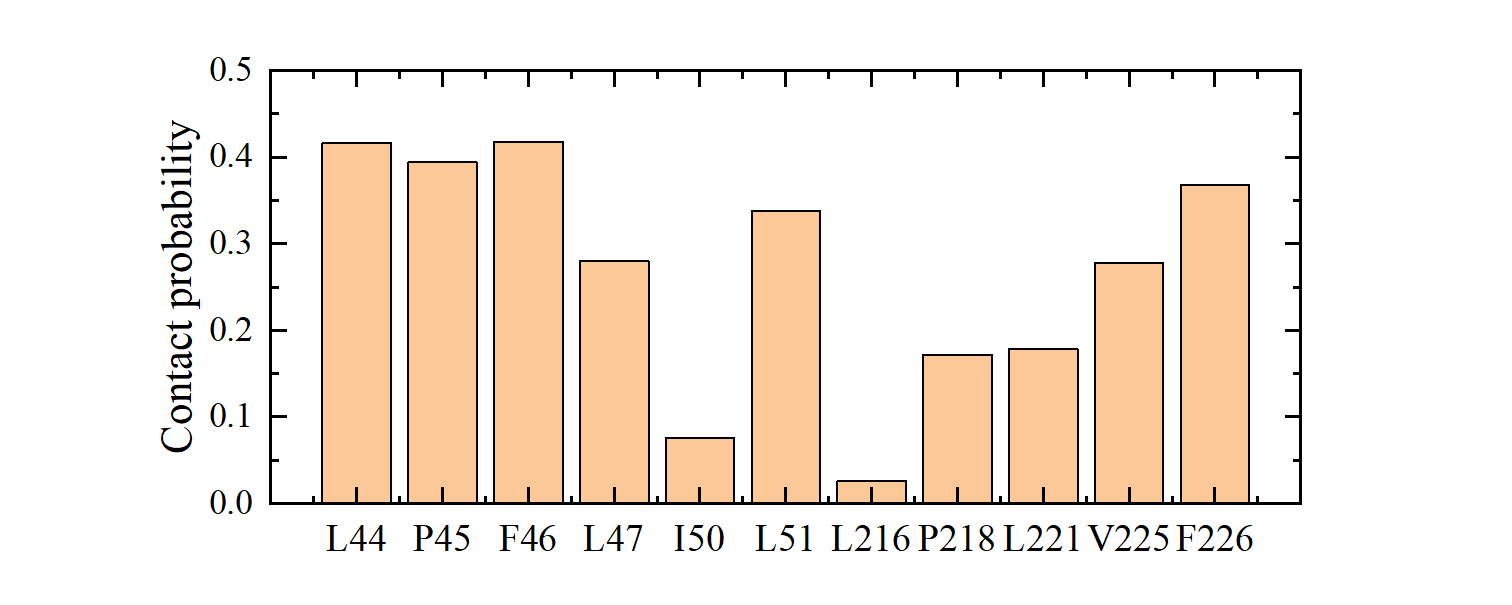


Figure S5. Contact probability of GQD to those residues associated Channel 2a.


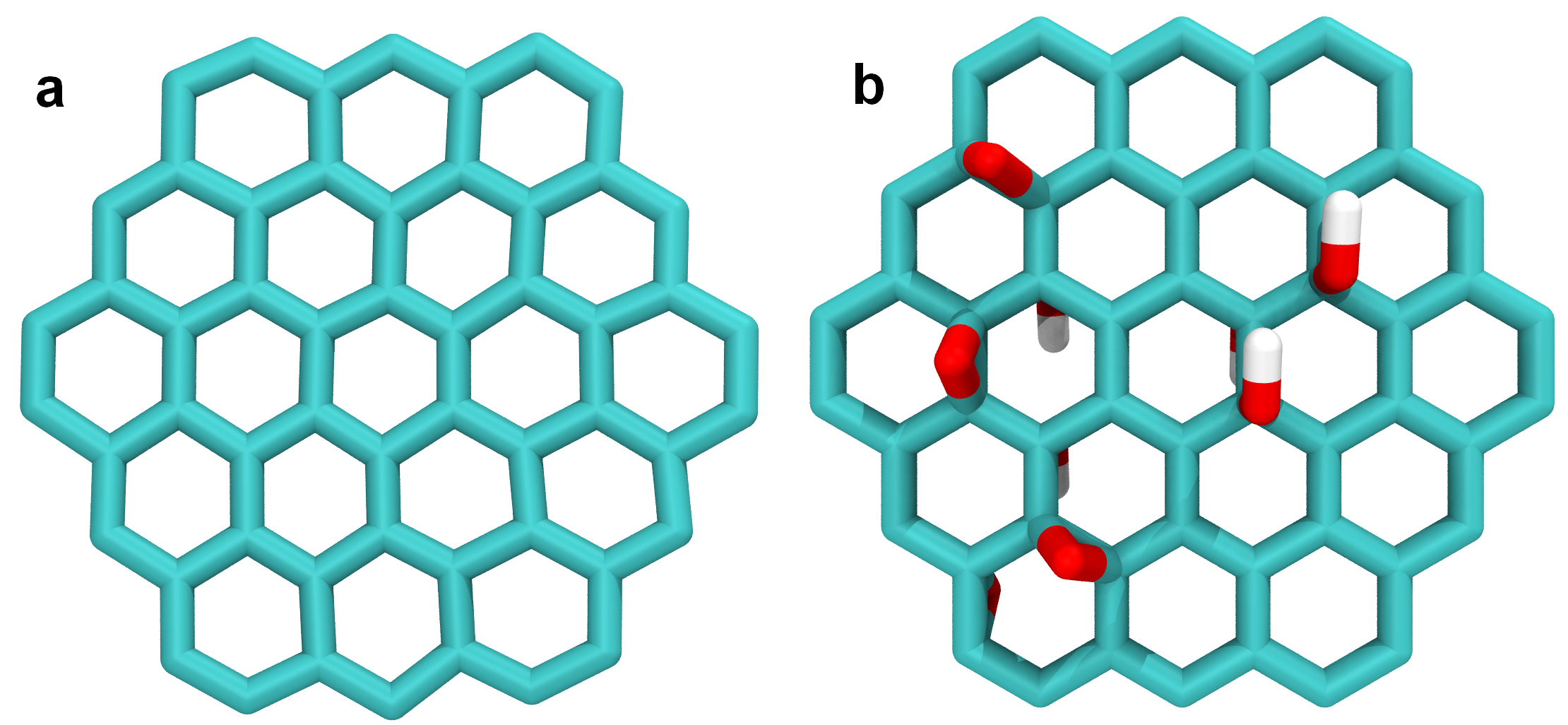


Figure S6. Configurations of GQD (a) and GOQD (b). Carbon, oxygen and hydrogen are shown with cyan, red and white colors, respectively. All the figures are generated by VMD software package (http://www.ks.uiuc.edu/Research/vmd/, J. Mol. Graph. Model. 1996, 14 (1), 33-38).
